# Complementary medicine use among Australian patients in an acute hospital setting: an exploratory, cross sectional study

**DOI:** 10.1186/s12906-019-2788-x

**Published:** 2019-12-21

**Authors:** Freya Waddington, Jenny Lee, Mark Naunton, Greg Kyle, Jackson Thomas, Gabrielle O’Kane

**Affiliations:** 1 Room12C21, University of Canberra, ACT, Bruce, 2615 Australia; 2 Queensland University of Technology, QLD, Brisbane, 4000 Australia

**Keywords:** Complementary medicine, Hospital, Prescribing, Pharmacy, Pharmacist

## Abstract

**Background:**

The use of Complementary Medicines (CMs) has significantly increased in Australia over the last decade. This study attempts to determine the extent to which complementary and alternative medicines are recorded, ceased or initiated in the acute hospital setting and investigate which health professionals have a role in this process.

**Methods:**

A cross-sectional study of inpatients was conducted at a major tertiary teaching hospital. Patient’s medical records were examined to determine the rates of complementary medicine (CM) use and recording on medication charts and discharge prescriptions. Patient progress notes were audited to determine which health professionals were involved with the initiation or cessation of CMs during the inpatient stay.

**Results:**

Three hundred and forty-one patients were included for analysis of which 44.3% (*n* = 151) participants were recorded as utilizing a CM. Patients were admitted on a mean of 2 (±1.4[Sd]; 0–9[range]) CMs and discharged on a mean of 1.7 CMs (±1.3[Sd]; 0–5[range]). 274 individual CMs were recorded on inpatient medication reconciliation forms with multivitamins, magnesium, fish oil and cholecalciferol recorded the most frequently. One hundred and fifty-eight changes to patient CM usage were recorded during the patient hospitalisation. One hundred and seven of these changes (68%) were not accounted for in the patient progress notes.

**Conclusion:**

Patients use of CM in this hospital setting do not reflect the national estimated usage. On the occasions that CM products are included in patient records, they are subsequently deprescribed following patient examination in hospital. It is currently unclear which health professionals have a role in this deprescribing process.

## Background

Complementary medicines (CMs) include vitamin, mineral, herbal and homeopathic products. The use of CMs has significantly increased in Australia over the last decade [1]. Many Australians invest heavily in CMs, with estimates suggesting spending on these products totals at over $3.5 billion (AUD) annually [1]. In a national study published in 2007, a survey found that complementary medicine use has increased with 68.9% of Australians having used at least one form of complementary medicine in the preceding year [2].

This increase has been attributed to people’s desire to seek alternative ways to improve their health and well-being, relieve symptoms associated with chronic ailments and to reduce side effects from conventional therapies [3]. CMs are often perceived by the public as “naturally derived plant products”, which are safer and have fewer side effects than traditional medicines [4, 5]. However, many CMs have been associated with severe adverse effects or interact dangerously with other CMs and/or prescription medications through affecting metabolism or interactions with transport proteins [6]. Recent research has provided an overview of the many potential interactions between CMs and prescribed drugs [7]. Many of these potential interactions bare relevance in the hospital setting. For example, commonly used CM products such as ginkgo and fish oil have been shown to effect bleeding and are therefore a necessary consideration for perioperative risk [8].

Although a large percentage of the Australian population appears to be utilising CMs, whether these products are accurately recorded, initiated or ceased in the hospital setting in Australia remains largely unknown [9–11].

Hospital pharmacists work within a multidisciplinary healthcare team, performing tasks that contribute to the medicines management pathway [12]. These tasks include medication reconciliation, review of medication usage, organized supply of medicines and the provision of medicine information to patients and other health professionals [12]. These services identify the risks associated with medicine use; reduce adverse drug events that consequently reduce the duration of hospital stay and burden to the economy [12]. Pharmacists are therefore ideally placed to assist in the recording of CM use of hospital inpatients through use of the medication reconciliation process. However, anecdotal evidence has suggested that CMs are commonly overlooked in this process. Despite the widespread use of CMs nationally, the literature suggests that health professionals do not consistently record CMs as a routine component of inpatient medication history, nor on inpatient medication charts [11]. Subsequently, there is an increased potential for misreported and potentially unsafe use of CMs in the hospital setting.

To date, there are limited studies that have investigated the recorded use of CMs in Australian hospital inpatients. In 1994, a study by Kristoffersen et al. conducted at Sydney’s Royal North Shore Hospital reported 52% of patients had utilized CMs in the 12 months preceding their hospital admission [9]. Further findings included that only 21% of patients informed their doctor of their CM usage [9]. Reasons included patients felt that doctors lack receptiveness to these therapies and also that patients felt a sense of autonomy and did not believe this medication use was relevant to the doctor [9].

A similar study by Welch was conducted in St Vincent’s Hospital in Sydney over a 3 week period in May 2000 [10]. This study found 12% of inpatients recorded utilizing complementary medicines at the time of their admission, and of these, 18% of patients were concurrently taking conventional medicines that could potentially interact with CMs at the time of their admission [10]. Welch concluded that use of CMs by patients admitted to hospital appeared significant and with the existing potential for drug interactions, routine inclusion of these medicines in the medication chart was necessary [10].

Finally, research published in 2005 by Cockayne et al., investigated the completeness of documentation of CM use in hospital charts before and after implementing an education program to staff [11]. This study found that although patient’s usage of CMs was recorded at 58% in the month prior to admission, only 28% of these CMs were documented in the medical records [11].

There is a need for strategies such as education of health professionals which may be beneficial in increasing awareness of the risks associated with some of these products and the need to record CM use. Previous research appears to have illustrated that documentation of CMs in the hospital may be suboptimal. The identification of these products in a patient’s medication history has the potential to enable health professionals to address the possible unnecessary or unsafe use of CMs by a high percentage of patients.

The aim of this research was to determine whether CMs are being recorded in hospital inpatient records at rates that reflect the previous literature and national estimates of usage, and, to determine the role of the health professionals, in particular, the hospital pharmacist, in this process.

## Methods

The study received ethical approval from the Australian Capital Territory (ACT) Health Human Research Ethics Committee (ETHLR.16.163). This research is a cross-sectional study which was conducted at a major tertiary teaching hospital in the Australian Capital Territory. Patients were assessed if they were inpatients from any ward and had been discharged from the hospital during a two-week period in June 2016 and were over 18 years of age, and were excluded if their inpatient stay was less than 5 consecutive days during this period. This admission time frame was implemented to allow suitable time for the medication reconciliation process to occur, and the patient to be considered by the wider multidisciplinary health professional team.

Each patient’s medical record was examined by one of two allocated researchers (JL or FW) to determine if the use of CMs was recorded both on the medication reconciliation form and on the inpatient medication chart. Consistency of collection was ensured between researchers through an agreed process of recording and a comparison of a random selection of results to detect any discrepancies. Nil discrepancies were identified in data collection. Medication reconciliation forms and inpatient medication charts were further scrutinised to determine product type and dose and if these products were either being initiated or ceased in the hospital setting. Further, corresponding patient discharge summaries were examined to determine the rates of inclusion of CMs on the discharge prescription. On the occasions that CMs were ceased during the inpatient stay, progress reports were audited by the corresponding researcher (JL or FW) to determine whether a clinical pharmacist or other health professional was involved during this process and had subsequently made note. Similarly, on the occasions that CMs were commenced during the inpatient stay, progress notes were also reviewed to determine which health professional was involved in this process.

CMs identified in the study were crosschecked with the Natural Medicines Database for ingredient type and categorised into multivitamins, minerals, vitamins, supplements, herbal and probiotic groups based on the product’s active ingredients.^10^

All data were analysed through use of IBM SPSS (version 24; IBM SPSS, Chicago, IL, USA) software. T-tests were conducted to determine any significance in changes in CM prescribing. A *p*-value of 0.05 was used to determine significance.

## Results

### CM usage

**Fig. 1:**
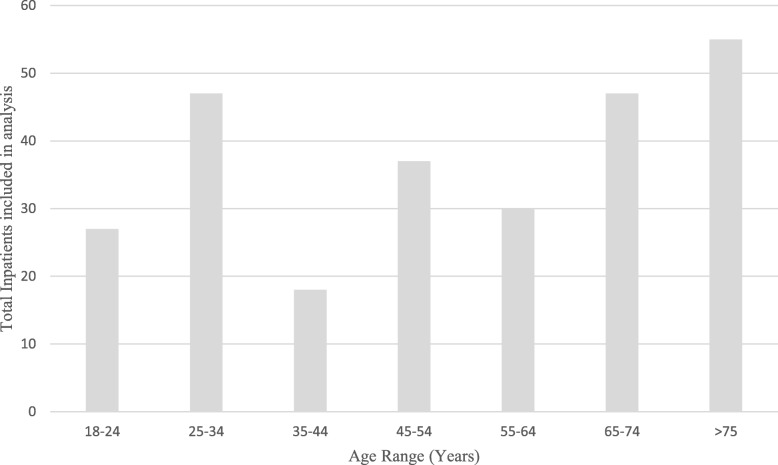
Age range of inpatients included for analysis

A total of 426 patient files were assessed; 85 (20%) were excluded from further analysis as either no medication reconciliation forms were completed during their stay, the patient was aged less than 18 years, the patient’s inpatient stay was less than 5 days or the patient deceased during their inpatient stay. One hundred and eighty males (53%) and 161 females (47%) were included in this study. The mean age of patients was 65 years (±19.1[SD; 18–94[range]) (Fig. 1). Participants were recorded as taking a mean of 7 regular and ‘as required’ medications (±4.9; 0–26) when admitted to the hospital, with a mean of 6 (±4.2; 0–25) of these medications taken via the oral route.

### Patient demographics

Of the 341 patients included in the analysis, 44% (*n* = 151) participants were found to have a CM recorded on their medication reconciliation form (see Table 1.). Of these, 54% (*n* = 82) were females and 46% (*n* = 69) were male patients. Patients aged over 75 years were the most frequent users of CMs with 55% (*n* = 71; 8[mean]; ±3.9[SD]; 0–19[range]) of inpatients in this age bracket recorded taking a CM. Patients aged between 35 and 44 years were found to have the lowest percentage of recorded CM use at 18% (*n* = 3; 2; ±1.7; 0–11).

**Table 1:** Demographics of included patients

Sex	
Male (%)	180 (53)
Female (%)	161 (47)
Age (Years)	
Mean (SD)	65 (19)
Medications	
Mean medications on admission (SD)	7 (4.9)
Mean CMs on admission (SD)	1 (1.5)
Mean CMs on discharge (SD)	1 (1.2)
Length of Hospital Stay	
Mean days (SD)	1 (1.6)
Patients with CM recorded on Medication Reconciliation Form	
Male (%)	69 (46)
Female (%)	82 (54)

**Table 2:** Frequency of health professional’s recorded recommendations for initiating or ceasing CMs during the inpatient stay in the study cohort

Health Professional Type	No. of CMs Initiated	No. of CMs Ceased	Total Changes to CMs
Unknown	55	52	107 (67.7%)
Doctor	31	7	38 (24.1%)
Dietitian	7	0	7 (4.4%)
Pharmacist	2	3	5 (3.2%)
Nurse	0	1	1 (0.6%)

Of the patients admitted to hospital found to be taking CMs, the mean number of CMs taken was 2.3 (±1.4[SD]; 0–9[range]). This same population of patients was subsequently found to be discharged on a mean of 1.7 CMs (±1.3; 0–5). Of those individuals admitted to hospital whilst taking a CM, 35% (*n* = 52) were taking a single CM.

### Type of CMs utilised by patients

**Fig. 2:**
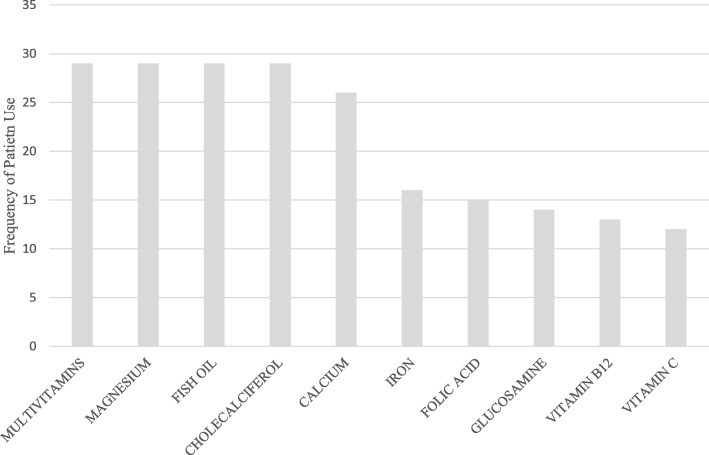
Ten most frequently utilized complementary medicines

A total of 274 individual CMs were recorded on inpatient medication reconciliation forms. Multivitamins, magnesium, fish oil and cholecalciferol were identified as the most frequently recorded individual CMs, with each item was recorded on 29 (11%) occasions. Following categorization by ingredient type, single vitamin and single mineral medicines were found to be the most commonly utilised group of CMs. The 10 most frequently utilized CMs are displayed in Fig. 2.

### Initiation and cessation of CMS

A total of 158 changes to patient CM usage pattern were recorded during the patient inpatient stay. There was no documentation of 107 of these changes in the patient progress notes (see Table 2). Doctors were involved in making the highest number of recommended changes to patients’ CM usage, with 38 recommendations documented, this was followed by hospital dieticians (*n* = 7) and pharmacists (*n* = 5). These changes were inclusive of recommendations to initiate and/or cease the CM intake. On one occasion, a recommendation for cessation of a CM was found to be recorded by a nurse.

## Discussion

We identified that 44% of patients were using CM with females over the age of 75 years being the most frequent users. This is the first study in Australia to investigate the recording of CMs in inpatients in the hospital setting with a focus which health professional recorded patient CM use including the initiation and deprescribing of these agents, initiated or ceased in the hospital setting.

### CM usage

The results of a National Australian Population-Based Survey conducted by Xue et al. (2007) found the most common demographic of CM users were females aged between 18 to 34 year [2]. Similarly, a South Australian study found females aged between 25 to 44 years to be the most frequent users of CM [13]. Our results found higher usage of complementary medicine products to be recorded in much older women (i.e. > 75 years; 60%). These results therefore do not accurately reflect previously recorded national rates.

Further, our study revealed that 44% of examined inpatients had CM recorded in their medical notes, which is lower than the data presented by Cockayne et al. (2005) who found 58% of inpatients used CM and Xue et al. (2007) who reported approximately 69% of the Australian population used CM) [2, 11].

### Type of CMs utilised by patients

Currently, the most commonly recorded CMs used by Australians are single ingredient vitamins [14]. This is consistent with our study findings, which also revealed single ingredient vitamins (31%) to be the most frequently utilised products. The Australian Bureau of Statistics recently estimated the most frequently used complementary medicines products, these included multivitamins or multiminerals, single vitamin and mineral and supplements [14]. Our results are consistent with these statistics with multivitamins, magnesium, fish oil, cholecalciferol, vitamin C and glucosamine all found to be the most frequently utilized CMs in our study cohort. However, further studies are required to determine the necessity of the CM products that were seen to be utilized in higher frequencies.

### Initiation and cessation of CMs

A key finding from this study was the low level of documentation with regards to commencing and deprescribing CMs. On closer inspection of the patient progress notes, pharmacists were less likely to document any changes in CM use than doctors. As in this hospital setting, phamracists are unable to cease medications, this could be theorized to be due pharmacists verbally communicating suggested changes and relying on doctors to record actual changes in notes. The majority of modifications to CM prescriptions (68%) were unable to be allocated to an individual health professional as no documentation was made in the patient progress notes. These low rates of documentation regarding CMs suggest that health professionals may deem the documentation of changes regarding these products less important than other medical issues.

Of the patients admitted to hospital recorded as taking CMs, the mean number of CMs taken was 2.3 products per individual. The same population of patients was subsequently found to be discharged on a mean of 1.7 CM products per individual. This illustrates that some of these CMs are in fact being ceased in the hospital. Health professionals may have recognized the potential issues relating to polypharmacy resulting from unnecessary use of CMs. Polypharmacy is appropriate in many individuals; however, when the benefit of its use is outweighed by its risk, polypharmacy should be minimised [15]. Patients who are prescribed with multiple medications have been shown to be at greater risk of non-adherence, hospitalization, adverse drug reactions, morbidity and mortality [15, 16]. Many CMs have been shown to provide little health benefit to patients and therefore cessation of these products is likely to be appropriate and result in improved health outcomes [17]. Health professionals may have recognized that these products were unnecessary and/or potentially harmful. However, due to the low levels of documentation of changes relating to complementary medicines in the patient’s medical notes, it is not clear which health professionals are making these changes.

Although not investigated directly in this research, thorough recording of patient’s medication history is necessary to identify the potential drug interactions or unnecessary continuation of CMs. Pharmacist-led comprehensive medication reconciliations have been shown to provide a more accurate estimation of CM use in certain patient groups [18]. Further to this, pharmacist-led medication interventions reduce adverse events and have a proven economic value for reducing medication errors post discharge [19]. Previous research by Cockayne et al. (2005) illustrated that the implementation of a short-term education program of health professionals may result in better medication history taking that is more inclusive of CM products [11]. Subsequently, specialized short-term education of hospital pharmacists may increase documentation and reduce adverse events.

### Limitations

Limitations of this study include the single center nature of this investigation. A broader data collection strategy including multiple sites with varying population characteristics may allow larger data sets and more conclusive findings. This study also did not have access to demographic information that may influence patients’ use of CMs, these included ethnicity, income and education status. Further, patient’s reasons for utilizing CMs were not explicitly identified; however this has already been well established in previous studies [13].

The inconsistency between the results of this paper and previously recorded numbers may be attributed to the single center nature of this study which has only investigated a specific demographic. The tertiary hospital in which this study took place is located in the Australian Capital Territory (ACT) and services a population generally recognized as highly educated, and higher income. This may equate to more informed decision making among study participants, who do not appear to rely heavily on CM products. However, previous research has suggested that higher levels of education may result in higher usage of CM products [2]. Further to this, previous data by the Australian Government revealed that the residents from the ACT are more likely to use herbal preparations than other residents elsewhere in Australia [14]. ACT residents are reported to have a higher amount of disposable income, therefore it may translate to increased expenditure on CM products [20]. Subsequently, it is suggested that CMs are not being accurately documented during the hospital admission process.

## Conclusion

In this hospital based study, the CMs usage data do not accurately reflect the national estimated usage. It appears that on the occasions that CM products are included in patient records, they are subsequently deprescribed following patient examination in hospital. However, with the majority of the changes to inpatient CM prescriptions not documented, we are unable to determine the role the clinical pharmacist plays in this process. These results signal the need for an educational intervention targeted at pharmacy staff within the hospital to emphasize the importance of recording CM history in all patients is clear.

## Data Availability

The datasets used and/or analysed during the current study are available from the corresponding author on reasonable request.
